# Benefit sharing: an exploration on the contextual discourse of a changing concept

**DOI:** 10.1186/1472-6939-14-36

**Published:** 2013-09-12

**Authors:** Bege Dauda, Kris Dierickx

**Affiliations:** 1 Centre for Biomedical Ethics and Law, Faculty of Medicine KU Leuven, Kapucijnenvoer 35, Box 7001, Leuven B-3000, Belgium

**Keywords:** Benefit sharing, Research ethics, International research, Common heritage of humankind, Biodiversity, Justice, Developing countries

## Abstract

**Background:**

The concept of benefit sharing has been a topical issue on the international stage for more than two decades, gaining prominence in international law, research ethics and political philosophy. In spite of this prominence, the concept of benefit sharing is not devoid of controversies related to its definition and justification. This article examines the discourses and justifications of benefit sharing concept.

**Discussion:**

We examine the discourse on benefit sharing within three main spheres; namely: common heritage of humankind, access and use of genetic resources according to the Convention on Biological Diversity (CBD), and international clinical research. Benefit sharing has change from a concept that is enshrined in a legally binding regulation in the contexts of common heritage of humankind and CBD to a non-binding regulation in international clinical research. Nonetheless, there are more ethical justifications that accentuate benefit sharing in international clinical research than in the contexts of common heritage of humankind and the CBD.

**Summary:**

There is a need to develop a legal framework in order to strengthen the advocacy and decisiveness of benefit sharing practice in international health research. Based on this legal framework, research sponsors would be required to provide a minimum set of possible benefits to participants and communities in research. Such legal framework on benefit sharing will encourage research collaboration with local communities; and dispel mistrust between research sponsors and host communities. However, more research is needed—drawing from other international legal frameworks, to understand how such a legal framework on benefit sharing can be successfully formulated in international health research.

## Background

The concept of benefit sharing has been a topical issue on the international stage for more than two decades, gaining prominence in international law, research ethics and political philosophy [1]. This prominence of benefit sharing is mostly accompanied with controversies and contradictions associated with what the concept entails and what its definition is. Schroeder realizes this problem of definition, commenting, “for more than 15 years of entering into international law, benefit sharing has no entry in the Oxford English Dictionary and remains a technical word not used in everyday academic language” [2].

Nonetheless, navigating through the contexts where benefit sharing is prominent, we can find a definition that suits the concept as used in that context. For example, in the framework of the access and use of genetic resources, benefit sharing is described as “the action of giving a portion of advantages or profits derived from the use of genetic resources or traditional knowledge to resource providers in order to achieve justice in exchange” [1]. In the context of international research this definition is viewed differently; it is often viewed from the perspective of what participants and by extension the communities in developing countries ought to receive as compensation for their participation in research [3].

Differences in the discourses and justifications on benefit sharing form the basis for different definitions and also limit or broaden the concept. For example, a compensatory justice-based discourse may limit benefit sharing to the compensatory interaction which occurs between researchers and participants during research, while solidarity-based justifications broaden benefits to include all human beings [3]. It is these discourses and justifications that set the tone for this article. This article provides an investigation into the changes in the discourses and justifications of benefit sharing in order to address the question of whether these changes affect the present practice of benefit sharing in international research. We examine these changes in three spheres. Firstly, we assess the discourse of benefit sharing in terms of the broader concept of the common heritage of humankind—we assess the emergence and the ethical disposition that benefit sharing concept is set to achieve in the common heritage of humankind. Secondly, we assess the concept in the context of the access and use of genetic resources as outlined in the Convention on Biological Diversity (CBD). Thirdly, we examine the concept as used in the context of international clinical research with a focus on three formulations of benefit sharing. This article will familiarize the reader on the main discourses on benefit sharing. It is intended to contribute to awareness among stakeholders in health research on benefit sharing.

## Discussion

We present an account of the three spheres on which benefit sharing is commonly discussed. Based on these spheres, we then map ethico-legal changes in benefit sharing and assess these changes as they affect benefit sharing in international research.

### Benefit sharing within the context of the common heritage of humankind

The notion of benefit sharing first emerged on the international platform in relation to the concept of the common heritage of humankind [4]. The common heritage of humankind is a concept that deals with the fate of resources obtained from common heritage territories (the moon and other celestial bodies, as well as the sea and the subsoil beneath it). The concept of the common heritage of humankind evolved from the doctrine of *res communis* which delineates that resources obtained from common heritage territories are not meant to be monopolized, possessed or owned by individuals, communities or states; rather, the use of such resources has to be subjected to the rights and interests of all humankind [5,6].

The relationship between the common heritage of humankind and benefit sharing stems from one of the cardinal elements outlined in the common heritage of humankind framework, namely: equitable sharing of resources. This cardinal element is closely related to benefit sharing [4]; it calls for an equal distribution of resources and encourages global policies that foster a homogeneous state of affairs among all states with respect to common heritage resources. Developing countries have envisaged this benefit sharing as a tool that presents a solution to the disparities existing between developing and developed states [7]. Hence, it has been advocated that the benefit sharing of the common heritage of humankind should be extended beyond the sharing of tangible resources to other possible goods [7]. For example, some scholars point out that benefit sharing should also include the sharing of technology with other states. This is based on the assumption that technology is a common heritage of humankind because it is an inheritance of our ancestors irrespective of their nationalities [8].

Notable international treaties that emphasize benefit sharing in their common heritage of humankind regimes include the United Nations Convention on Law of the Sea (UNCLOS) and the International Undertaking on Plant Genetic Resources (IUPGR). The UNCLOS stresses in article 140 paragraph 1 the need to share benefits to everyone irrespective of geographical location of states, whether coastal or land-locked. The Convention also indicates the need to particularly consider the vulnerability of developing states that may be at risk of exclusion from benefit distribution [9]. Also, plant genetic resources have been considered as part of the common heritage of humankind [4,10]. This was indicated by the agreement of International Undertaking on Plant Genetic Resources (IUPGR). The agreement recognizes that plant genetic resources are a heritage of humankind to be preserved, and to be freely available for use, for the benefit of present and future generations [11].

The ethical appeal of benefit sharing in the common heritage of humankind is targeted at achieving equality among all states with regards to resource distribution. The founder of the concept of common heritage of humankind, Arvid Pardo [12] clearly indicates this equality in a statement that: “the common heritage of humankind challenges the structural differences between rich and poor countries and revolutionizes international relations towards equality among countries” [13]. However, the reality of achieving equality of resource distribution remains problematic owing to the continuous power and economic asymmetry that exists among states. Mounting concern by developing countries about the uncompensated use of plant genetic diversity obtained from their territories pushed for a move against the common heritage of humankind concept and an adoption of sovereign rights to biodiversity [4]. It was argued that the common heritage of humankind and its benefit sharing requirements would encourage exploitation and biopiracy—a situation whereby bioprospectors travel to diversity rich countries and take resources without seeking permission or sharing benefits with host countries or local communities [14].

### Benefit sharing in the context of the access and use of genetic resources according to the Convention on Biological Diversity (CBD)

The concept of benefit sharing has been concretized in the context of the access and use of genetic resources [15]. Genetic resources include both non human genetic resources (e.g. plant, micro organism and animal genetic resources) and human genetic resources (e.g. human DNA material, blood and other human tissues). In this context, benefit sharing denotes an exchange between those who grant access to genetic resources and those who provide benefits, rewards or compensations resulting from the use of the genetic resources [1,14]. Unlike the common heritage of humankind concept, in the context of genetic resources, states hold a sovereign right over their natural resources and can grant access to those that require to utilize such resources under a condition of Prior Informed Consent (PIC) and Mutually Agreed Terms (MAT) of appropriate benefit sharing [16]. For example, a local community in the Amazon region of South America can negotiate a deal with plant scientists or bioprospectors to exchange plant material expected to have a medicinal property with medicinal products or royalties that subsequently result from the utilization of the plant material.

The concept of benefit sharing with regard to the use of genetic resources originates from the CBD. The CBD is said to mark the end of the era of the common heritage of humankind concept. In the CBD preambles, resources are deemed to be the “common concern” instead of the “common heritage” of humankind. It is the concern of all humankind to preserve and sustain the use of resources for humanity and future generations. However, the duty to preserve and sustain resources does not imply that resources are common heritage for all; rather, resources are the property of the states [10]. CBD declaration was first endorsed in 1992 however; the requirement of benefit sharing was fortified through a series of discussions by the Conference of Parties to the CBD which subsequently culminated into a more emphatic framework known as the Nagoya Protocol. The protocol which was adopted in 2010 provides a strong basis for greater legal certainty and transparency for both providers and users of genetic resources [17]. The legal certainties of the protocol require countries to develop laws to ensure that the use of genetic resources within their jurisdiction is done with prior informed consent and on mutually agreed terms, and that it complies with benefit sharing legislation in other countries. While the transparency aspect of the protocol require industrialized countries to set up one or more checkpoints for disclosing what resources they have accessed and where, and to monitor whether they are complying with the protocol [17]. In general, the Nagoya Protocol addresses some critical gaps and uncertainties in the CBD regulations on benefit sharing and also sets in motion formal discussions on other unresolved topics and ideas [18].

The concept of benefit sharing here can be related to the ethical principle of justice in exchange or commutative justice. Justice in exchange demands that those who use resources give back due reward to the providers or custodians of the resources [19]. This type of justice has been quoted in articles to be related to Aristotelian notion of fairness in a transaction which holds that the intrinsic worth of something has to be matched by a proportionate requital either in kind or in pecuniary terms [4,19]. While the ABS structure works for non-human genetic resources, human genetic resources could not be retained within the framework of the CBD. The Conference of Parties to CBD in 1995 excluded human genetic resources from the legal framework of its promulgation [20].

### Benefit sharing and international clinical research

International clinical research refers to a research involving human subjects that is organized and sponsored by pharmaceutical industries, Contract Research Organizations (CROs) and other research organizations in industrialized countries but conducted or outsourced in resource poor countries [21]. International clinical research can be clinical trials of new drugs, testing of new diagnostic equipment or genetic research that involves the collection and storage of various genetic samples e.g. Genome Wide Association Studies. Outsourcing of research by pharmaceutical industries has been on the rise and is mainly due to the ease of patient recruitment, low overall cost of trial and relatively less stringent regulatory procedures in the outsourced countries. These reasons raise concerns on how to conduct research that is ethical in resource poor countries [21,22]. Benefit sharing is considered to be one of the important benchmarks for ethical research in developing countries [23]. Also, the discourse on benefit sharing in international research springs from the fact that large proportion of the populations in developing countries lives in poverty and cannot pay for their health care services. As such, health research conducted in these resource poor countries should uphold obligations of providing the benefits of research in order to improve the healthcare services of these countries [24].

In human genetic research, the predominant stance on benefit sharing suggests a return to the concept of the common heritage of humankind. This is evident from the emphasis on an equal sharing of the benefits arising from human genetic resources to all of humanity. The conjecture in human genetic research is that human beings share 99.9% of their genome; as such, human genes are considered to be resources of the common heritage of humankind, and all humans ought to share the collective duty to explore their genetic resources, preserve them and share equally the benefits derived from their utilization. This position was declared by the United Nations Educational, Scientific and Cultural Organization (UNESCO) Declaration on Human Genome and Human Rights (1997) and followed by the Human Genome Organization (HUGO) Committee on Ethics (2000). The main ethical disposition regarding benefit sharing in human genetic research is centered on solidarity, whereby everyone (not only the research participants) is entitled to the benefits derived from advances in genetic research. This is evident in the UNESCO declaration which states that “benefits from advances in biology, genetics and medicine, concerning the human genome, shall be made available to all” [25]. To demonstrate the need for commitment to support developing countries through benefit sharing, the HUGO ethics committee proposed that 1-3% of the profits gained by research organizations should be set aside for charitable work in developing countries. Setting this figure was intended to provide a minimum requirement that would encourage companies to be good universal citizens [26].

Furthermore, benefit sharing in international research is viewed in three perspectives. Firstly, benefit sharing is viewed as the provision of post-research results of the proven intervention. In other words, when the intervention tested in research has shown to be effective, it should be made “reasonably available” to the host community. This so-called “reasonable availability” in research emerged during the debate on the standard of care to be accorded to research participants in developing countries [27]. Reasonable availability requires that research be tailored to the health needs of the host community and the research results be made available to the community at the end of the research [28,29]. This requirement is found in international ethical guidelines for the conduct of research. For example, the Helsinki declaration states in paragraph 33 that, “at the conclusion of the study, patients entered into the study are entitled to be informed about the outcome of the study and to share any benefits that result from it, for example, access to interventions identified as beneficial in the study or to other appropriate care or benefits” [30]. It is noteworthy to know that a new draft of the Helsinki declaration is formulated and is geared towards a development of a new version of the Helsinki Declaration—the draft has a recommendation for amendments on the wordings of reasonable availability and benefit sharing [31].

Reasonable availability can be seen in the light of justice as reciprocity. It is concerned with what people deserve as a function of what they have contributed to an enterprise or the society. In clinical research, justice as reciprocity means that something is owed to the research participants and the community even after their participation in a trial has ended, because it is only through their acceptance of risk and inconvenience that researchers are able to generate findings necessary to advance knowledge and develop new medical interventions [32].

Secondly, benefit sharing in international research is viewed through the lens of the fair benefit approach, whereby the research community and research sponsors enter into a bargain in order to derive what is appropriate and fair benefit(s) that the participants should accrue [33]. The fair benefit approach argues for proportionality of benefits with regard to the risks and burdens of participation in research. The higher the risk of participation in research, the higher the benefit accrued ought to be. The approach also delineates that fairness of benefit is concerned with how much benefit is given to the participants or community, not what type of benefit they accrue as in the case of reasonable availability [33]. The fundamental difference between the reasonable availability and fair benefit approaches is that while the former restricts benefits to the proven intervention only, the latter expands the range of benefits to other possible benefits besides the proven intervention. This difference is echoed by the Participants at the Conference on Ethical Aspects of Research: post-research intervention is one way to provide benefits to the community but not the only way, and it must not be considered a necessary ethical condition for research in developing countries [34].

Thirdly, benefit sharing can be viewed according to the maximin approach. Maximin places benefit sharing in a broader concept of global distributive justice and depart from the view that negotiating activities between parties (sponsors and participants or communities) should always favor the disadvantaged group in their benefit sharing formulations [35]. The maximin approach is suitable considering the marked power and economic asymmetry between research sponsors and the vulnerable research community. Such asymmetry makes the vulnerable population view research as an opportunity to access better healthcare or to improve their health conditions. As a result, research communities in developing countries that strive to access basic goods (e.g. healthcare) during research should be assured of some benefits in the spirit of global distributive justice for basic goods [27,36]. With regard to the fairness of benefit sharing, the maximin approach suggests that a threshold of benefits should be set, beyond which it would no longer be rational for a self-interested research sponsor to transact [35]. In more practical terms, maximin advocates that pharmaceutical industries that outsource research to poor communities provide the best deal of benefits so that the poor accrue more benefits from the excesses gained as a result of the research conducted in the poor community.

The main emphasis in the maximin approach is on benefits on a macro level—i.e. benefits that will improve the overall healthcare structures of the poor communities. This is in contrast to the fair benefit approach which relies on the procedural bargaining powers of the parties involved in the transaction [35]. The maximin approach is seen as a way in which general improvement in healthcare as a basic good can be obtained through research, and consequently the gap of health-related inequality between the rich and poor can be bridged. Furthermore, the maximin approach has a positive advantage in that it provides benefit to the community irrespective of whether a post-research product is developed or not from the research [37].

### Ethical and legal changes on benefit sharing

From the foregoing discussion, it is clear that benefit sharing has undergone ethical and legal changes from its inception in the common heritage of humankind concept to its usage in international research (Table 1). Regarding the ethical changes, we can ascertain that the main ethical justification of benefit sharing changes in each context. In the context of the common heritage of humankind, benefit sharing is justified based on the principle of justice as equality whereby everyone deserves the same dignity, respect and moral worth [38]. Justice as equality entitles every state as a matter of rights to the benefits derived from the common heritage of humankind and it would be unjust not to share equally the benefits arising from the utilization of these common heritage resources. This ethical disposition has changed in the premise of the CBD. The justification of benefit sharing in the CBD is fundamentally based on the principle of justice in exchange or commutative justice. This exchange is between states that provide genetic resources on the one hand and states that access/utilize such resources on the other hand. Justice in exchange demands that fairness is ensured in what is exchanged when states interact with each other [39]. Therefore it would be unjust for a state not to provide fair and equitable benefit for an exchange of plant or micro-organism genetic material given to her.

**Table 1:** Benefit sharing disposition in various contexts

**Context of benefit sharing**	**Main ethical justification**	**Parties involved in benefit sharing**	**Legal stance of benefit sharing regulations**
Common Heritage of Humankind	Justice as equality	All states of the universe	Binding regulations (e.g. UNCLOS 1982, IUPGR 1983)
Convention on Biological Diversity	Justice in exchange or Commutative justice	States that provide genetic resources	Binding regulations on states that ratified the CBD
		States that utilize genetic resources	
International research	Justice as reciprocity (reasonable availability)	Pharmaceutical industries and research organization	Non binding regulations
	Procedural justice (fair benefit approach)	Research participants and communities	
	Distributive justice (Maximin)		
	Solidarity (Genetic research)		

In the context of international research, there are four major justifications that benefit sharing is based on. Firstly, taking on the discourse of human genetic research, benefit sharing is justified on solidarity reasons whereby participants, communities and other populations outside the research setting have the right to benefits from the fruits of the research. In other words, benefit sharing is a gesture of solidarity between research sponsors on the one hand and participants, communities and in extension other populations on the other hand. As participants contribute their genes for research in solidarity, sponsors should return back this solidarity by distributing benefits to participants and everyone [40]. Secondly, justice as reciprocity also provides a justification for benefit sharing in international research considering the “reasonable availability” viewpoint. Participants’ contributions are reciprocated with products generated from research for their efforts, time and risks taken in research [33]. Thirdly, benefit sharing is also justified on the basis of procedural justice in international research. This is mainly seen in the fair benefit approach model [34]. Procedural justice is considered to be the main ethical disposition in the fair benefits approach because the approach emphasizes that fairness on benefit sharing is achieved when research participants and communities enter into a bargain or negotiation with the research sponsors in order to achieve fairness on benefit sharing—and the processes of negotiation or bargain must be made transparent to all. In other words as long as the negotiations between research sponsors and communities are transparent and the parties involved have reach an agreement, then the benefit sharing is said to be fair [34]. Fourthly, based on the maximin approach, benefit sharing is justified on the basis of global justice for health. In this case, background inequalities between research communities and research sponsors form the main reason for benefit sharing. On the basis of this health inequality, global justice demands that research benefits should not only target the small needs of the research community but also the large needs of basic healthcare of the community and strive for improved health systems [41]. In summary we can say that the main ethical justification of benefit sharing has changed from justice as equality in a concept of the common heritage of humankind to justice in exchange in the CBD and to four major justifications in the context of international research.

With regard to legal changes, benefit sharing has undergone a shift in terms of its force and protection under the law (Table 1). Under the common heritage of humankind concept, the concept of benefit sharing exerts protection as it is enshrined under a legally binding agreement. States that endorse the common heritage of humankind law have to agree with its accompanied benefit sharing regulation. This also applies to the CBD regulation on the use of non-human genetic resources. The CBD is even more stringent as it has a separate binding agreement on benefit sharing (Nagoya Protocol) which legally regulates non human genetic resources. However, with regard to the use of human genetic resources and in international research the protection of benefit sharing by a binding document is absent. This represents a shift away from a concept that is protected by law to a non binding regulation as documented in the UNESCO Declaration on the Human Genome and Human Rights (1997), the HUGO Ethics Committee statement on benefit sharing (2000), the UNESCO Declaration on Bioethics and Human Rights (2005) and the Helsinki Declaration of the World Medical Association (WMA, 2008).

The question of importance hitherto is: do the changes in the ethical and legal stance on benefit sharing in international research affect the present practice of benefit sharing in international research? One can consistently hold the view that making a certain regulation into law gives it supremacy and makes it firm in terms of implementation among states [42]. In other words, regulations that are passed into law are meant to be taken seriously and states are obliged to abide to the regulations whether they suit their intentions or not. The law therefore is always devised to enforce a duty without consideration of individual choices. We can also assert that some laws are formulated from certain ethical features that are deemed fundamental to societal living and human co-existence. For example, the rich biodiversity in developing countries is important to human existence—the need to protect and sustain its utilization for future generation is of immense significance, hence the CBD law created to protect against uncontrolled use and biopiracy of the biodiversity [19]. It goes without saying that if benefit sharing in international research is to be taken seriously as a vital substance of global health research, then its regulations must be enshrined into a legally binding framework. The absence of a legally binding document to regulate benefit sharing in international research has undoubtedly affected the tenacity of its advocacy in practice.

## Summary

Based on the account of the ethico-legal changes, we suggest that benefit sharing in international research be formulated into a legal framework, as this will underscore the need to take it more seriously. Benefit sharing should be formulated in such a way that the level of benefits accrued from research participation is correspondingly matched with certain parameters such as the type of research in question, the organization sponsoring the research, the purpose of the research etc. In other words, a map of different possible types of research should be made and this should be matched with minimum forms of possible benefits that correspond to the research in question. The research sponsors can provide more than this minimum form of possible benefits if they are willing, however the minimum standard of benefits are obligatory by law. For example, a sponsor collaborating in a malaria vaccine research with a community should provide a range of benefits—for instance, environmental fumigation services, distribution of insecticide-treated nets to members of the community, effective malaria treatments for participants that may develop malaria during research etc. This range of benefits would be considered to be the minimum standard that must be provided to the collaborating community. The collaborating research community can negotiate with the research sponsors for other forms of benefits they may prefer. All the procedures and agreements on research benefits between the sponsor and community must be properly documented and be legally binding. This legal framework is necessary because benefit sharing is mostly ignored even though it is regarded as an ethically sound concept in international research [43]. The legal framework will ensure that international research actors abide at all times to the set legal requirements of benefits whenever they interact with research communities. Furthermore, because benefits will be linked to legal promulgation, the local research community will be encouraged to collaborate and be more open in research because they are certain of benefiting from the fruits of research. A legal framework of benefit sharing will dispel the issue of mistrust between research sponsors and host states or communities where research is done. An example of such mistrust is seen in the recent case of the Indonesian government’s refusal to provide H5N1 samples from its citizens to the international research community for vaccine development on the grounds that they are not sure if the benefits of such a vaccine would be shared fairly with the Indonesian people [44].

While we hold the view that a non binding stance of benefit sharing affects its current standing in international research, we do not believe that the multiple ethical justifications in international research have also affected the stance of benefit sharing. The multiple justifications provide different ethical platforms on which the normative bearings of benefit sharing can be ascertained. As such, the multiple justifications only provide different pathways to benefit sharing formulations and do not weaken the normative ethical appeal of the concept, as Simms rightly puts: “the existence of various arguments [justifications] behind benefit sharing is not necessarily problematic in itself […] the justifications can however produce different benefit sharing rationale” [3].

We must affirm however, that while a legal framework of benefit sharing in international research would augment its practice, the ease of formulating such a framework is not a simple task. More research is needed in order to determine factors that will facilitate creating a benefit sharing law in international research. Research is needed to establish lessons from the enactment of CBD laws that will foster the development of a legal benefit sharing framework in international research. There is a need to critically ascertain other international legal frameworks that directly or indirectly affect the practice of benefit sharing e.g. the Intellectual Property Laws. Researches on different contexts, various stakeholders as well as the complexities of international research are needed to establish different forms of benefit sharing formulations that are feasible. Different benefit sharing formulations can be related to the type of research in question. This will help in establishing fairness of benefit with regard to the risk of research. Also, more justifications and motivations for benefit sharing among international research sponsors need to be investigated.

## Abbreviations

CBD: Convention on biological diversity; CROs: Contract research organizations; HUGO: Human genome organization; IUPGR: International undertaking on plant genetic resources; MAT: Mutually agreed terms; PIC: Prior informed consent; UNCLOS: United Nations convention on law of the sea; UNESCO: United Nations educational, scientific and cultural organization.

## Competing interests

The authors declare that they have no competing interests.

## Authors’ contributions

The article was developed at various stages of drafted manuscript. BD and KD contributed equally to the first draft. BD elaborated the various stages of the manuscript with thorough revision, editing and mentoring from KD during the pre-publication process. Both authors read and approved the final version of the manuscript.

## Pre-publication history

The pre-publication history for this paper can be accessed here:

http://www.biomedcentral.com/1472-6939/14/36/prepub
